# Is the choice of the statistical model relevant in the cost estimation of patients with chronic diseases? An empirical approach by the Piedmont Diabetes Registry

**DOI:** 10.1186/s12913-015-1241-1

**Published:** 2015-12-30

**Authors:** Eva Pagano, Alessio Petrelli, Roberta Picariello, Franco Merletti, Roberto Gnavi, Graziella Bruno

**Affiliations:** Unit of Cancer Epidemiology, “Città della Salute e della Scienza” Hospital and CPO Piemonte, Turin, Italy; Epidemiology Unit, ASL 5, Piedmont Region, Grugliasco, Turin, Italy; National Institute for Health, Migration and Poverty (INMP), Rome, Italy; Department of Medical Sciences, University of Turin, corso Dogliotti 14, 10126 Turin, Italy

**Keywords:** Lifetime costs, Chronic diseases, Diabetes Mellitus, Modeling techniques

## Abstract

**Background:**

Chronic diseases impose large economic burdens. Cost analysis is not straightforward, particularly when the goal is to relate costs to specific patterns of covariates, and to compare costs between diseased and healthy populations. Using different statistical methods this study describes the impact on results and conclusions of analyzing health care costs in a population with diabetes.

**Methods:**

Direct health care costs of people living in Turin were estimated from administrative databases of the Regional Health System. Patients with diabetes were identified through the Piedmont Diabetes Registry. The effect of diabetes on mean annual expenditure was analyzed using the following multivariable models: 1) an ordinary least squares regression (OLS); 2) a lognormal linear regression model; 3) a generalized linear model (GLM) with gamma distribution. Presence of zero cost observation was handled by means of a two part model.

**Results:**

The OLS provides the effect of covariates in terms of absolute additive costs due to the presence of diabetes (€ 1,832). Lognormal and GLM provide relative estimates of the effect: the cost for diabetes would be six fold that for non diabetes patients calculated with the lognormal. The same data give a 2.6-fold increase if calculated with the GLM. Different methods provide quite different estimated costs for patients with and without diabetes, and different costs ratios between them, ranging from 3.2 to 5.6.

**Conclusions:**

Costs estimates of a chronic disease vary considerably depending on the statistical method employed; therefore a careful choice of methods to analyze data is required before inferring results.

## Background

Chronic diseases impose a large economic burden on the individual, national healthcare systems, and countries [1]. Awareness of such economic burden has led to a sharp increase in the number of studies on economic issues related to chronic diseases [2]. However, it should be emphasized that the estimation of mean patient cost is not straightforward, particularly when the goal of the analysis is to relate costs to a specific pattern of covariates, and to compare costs between people with and without the disease [3].

The following criticisms often characterize the medical cost data distribution:

- Skewness, due to the presence of a minority of subjects with higher medical cost compared with the rest of the population. This cannot be bypassed excluding the outliers from the analyses, as errors or looking to more robust measures such as the median value. Indeed, all observations are of interest in the decision-making process, providing additional information on health care service utilization and related costs of subgroups of the examined population [4];
- Presence of zero-cost observations due to the lack of treatment in some subgroups of the analysed population. Indeed, people with positive and those with zero-costs are likely to have different behaviour patterns in relation to the covariates, such as age comorbidities, socioeconomic level and access to health care services [5, 6];
- Presence of censored data, which do not allow to observe the subjects’ costs over the entire period of interest, mainly life-time span or the study follow up. Such censoring does not usually satisfy the condition of being independent and not informative, so that individuals who remain under observation are generally not representative of the population at risk in each group [7, 8]; and
- Clustering of data, that is the presence of correlation between costs and outcomes. Indeed, clinical practice differs according to the centre or the general practitioner and patient case-mix [9–11].

Both the afore mentioned issues and methodological approaches for their handling have been widely debated in literature. Several appropriate statistical methods are now agreed upon and recommended [3, 12, 13]. In spite of a large amount of papers on costs of chronic diseases published in clinical journals, most of the critical remarks have appeared in the health economics journals and statistical literature. Therefore, we believe that introducing these issues to an audience of clinicians might allow them to better appreciate the implications of appropriate modelling of cost data on results and final decision-making.

The present study describes the impact of analyzing health care costs on results and conclusions in a population affected by diabetes using different statistical methods. Our data are affected by skewness and relevant presence of zero cost observations, so the focus of our paper will be on these two criticisms and how to manage them using different statistical models.

## Methods

### Data

Diabetes patients were identified through the Piedmont Diabetes Registry among the residents in the North-Western Italian city of Turin (population: 896,914). The Registry is based upon anonymous record linkage between administrative databases, lists of exemptions from payment of drugs, hospital discharge records and prescription databases. Details on the identification of the population-based cohort are described elsewhere [14, 15]. Italian citizens, irrespective of social class or income, are cared for by general practitioners and health care services are supplied by the National Health System (NHS). All drug prescriptions, outpatient treatment, diabetes related prescriptions of medical devices, such as test strips, syringes, and glucometers, hospital discharges and emergency room admissions are recorded by the Regional NHS Administrative Databases. Data registered from August 1st, 2003 to July 31st, 2004 were linked to the overall Turin population, making it possible to analyze health care services used by patients with and without diabetes (respectively *n* = 33,792 and *n* = 863,122). As previously described, we analyzed reimbursement tariffs set by regional and national government contracts [14]. In the present study, data were used for tutorial purpose only and for their distributive characteristics. An update of the data was not included in the study aims.

The Piedmont Diabetes Registry is authorized to use administrative health care data for epidemiological purposes. Raw anonymous data are available upon request to the Authors.

### Cost analyses

Effect of diabetes on mean annual NHS expenditure was analyzed over the entire cohort with several multivariable models, adjusted for age and gender.

First, we fitted one part models (Table 1), including: 1) an ordinary least squares regression (OLS); 2) a lognormal linear regression model; and 3) a generalized linear model (GLM) with gamma distribution.

**Table 1:** Determinants of annual healthcare costs, mean annual predictions and cost ratios (patients with vs. without diabetes), Root Mean Squared Errors (RMSE), by several data modeling approaches

Model		Diabetes (CI 95 %)		Cost (€) per person/year, patients with diabetes (*N* = 33,792)	CI 95 %^a^	Cost (€) per person/year, patients without diabetes (*N* = 863,122)	CI 95 %^a^	Cost Ratio (with vs. without diabetes)	RMSE
One-part models									
	*Normal (€)*	1,832.76	1,795.56–1,869.95	3348.6	3343.8–3353.9	831.2	829.8–832.4	4.03	3,342.4
	*Lognormal* ^*b*^ *(exp β)*	6.0	5.84–6.16	6146.5	6116.9–6178.6	1343.6	1340.5–1347.0	4.57	3,670.0
	*Gamma (exp β)*	2.6	2.56–2.67	3878.1	3867.0–3891.1	826.1	824.8–827.3	4.69	3,351.1
Two-part models									
Part 1									
	*Logistic (OR)*	2.40	2.18–2.64					-	
Part 2									
	*Normal (€)*	1,710.36	1,668.40–1,752.32	3392.0	3387.2–3397.5	1058.8	1057.4–1060.4	3.20	3,732.2
	*Lognormal (exp β)*	3.3	3.21–3.32	4119.9	4104.4–4136.3	1175.2	1173.2–1177.6	5.60	3,760.6
	*Gamma (exp β)*	2.2	2.21–2.28	3700.1	3690.0–3711.1	1050.8	1049.5–1052.4	3.50	3,735.6
Two part model (logistic + gamma)				3662.26	3652.07–3673.25	891.9	890.63–893.54	4.10	3,739.8

^a^derived by boostrapping method

^b^the log transformed outcome variable was (cost + 1)

The OLS model relies on the central limit theorem whereby the mean of a sufficiently large sample will be approximately normally distributed, independently of the population distribution. It assumes a linear relationship between the cost accumulation and its possible determinants (such as sex, age, type of diabetes etc.), with an additive effect of the covariates – that is the cost is a function of the Variable 1 effect plus the Variable 2 effect plus the Variable 3 effect, etc., − and a normal distribution of the error term. As OLS regression is well known and easy to apply, it is attractive for researchers and widely employed. However, in presence of skewness in the distribution of the error terms, OLS is not robust enough and can estimate inaccurate standard errors and confidence intervals. To overcome the problem of skewness in the residuals, a commonly adopted approach is to model a log-transformation of the response variable (that is, costs) able to gain a reasonable normalization effect even in presence of highly skewed data. To obtain results in natural units (euros, dollars), the approach of transforming the costs in any case requires a back-transformation at the moment of interpreting results. Such back-transformation might cause several additional problems, partially avoided by using specific statistical approaches (like the “smearing” estimator) [16]. In this analysis we applied the Duan smearing estimate [17], that is the average of the exponential of the residuals from the OLS regression on the log-transformed costs. If *c*_*i*_(*i* = 1, …, *n*) is the cost observed for each patient, and *x*_*j*_ are the *j*(*j* = 1, …, *h*) covariates and *β*_*j*_ are the *j* corresponding regression coefficients estimated with the OLS method, the smearing factor (Φ is:

$$ \varPhi =\frac{1}{n}{\displaystyle \sum_{i=1}^n \exp \left( \log \left({c}_i\right)-{x}_i\overset{\wedge }{\beta}\right)} $$

The exponentials of the predicted values were then multiplied by the smearing factor to obtain expected values on the original scale.

Moreover, in the present analysis the log transformed outcome variable was (cost + 1), as we needed to include all those subjects who had zero costs in the model also: in fact they could not be simply treated as missing cases, as they might convey relevant information on costs distribution among subgroups. It is common practice to add a constant to null values, when fitting log-linear regression models, in order to not exclude subjects from the analysis [12]. This is an arbitrary choice, that could bias the relationship between cost and covariates. However, sensitivity analysis shows that the distributions of original and transformed cost, stratified by the covariates used in the models, are substantially overlapped (data not shown).

The GLM models are a generalization of the linear model which specifies the relationship between a dependent variable and a set of predictor variables and allows the response variable to have other than a normal distribution [18, 19]. GLMs permit flexible modelling of covariates and enable inference to be made directly about the mean costs, rather than focusing on transformation methods. The relationship between the covariates and the mean of the dependent variable is described by the link function. The family specifies the distribution (such as normal, gamma, Poisson, etc.) that reflects the mean-variance relationship. As in most previous costs analyses, we used a Gamma distribution with a log link, that performs satisfactorily with distributions with zeroes and/or long right tails [13]. Lognormal and GLM are both multiplicative models, because they are expressed in logarithmic scale. This means that, due to the algebraic properties of logarithms, cost is a function of the exponential of the multiplied variables, after retransformation in the original scale. Consequently, the comparison of the estimated costs cannot be directly interpreted, due to the scale of the model and the technique of retransformation used.

In the second group of analyses (two part models, Table 1), the zero costs presence was handled by means of a two part model [20]: i) in the first part, a logistic regression was used to model the probability of incurring any cost over the one year period. The dependent variable was set equal to 1 in any subject who incurred costs, and was set equal to 0 in any subject who incurred in 0 costs. We also included covariates to adjust for age, sex and presence of diabetes. Odds ratios (ORs) for probabilities of using health services (i.e. of not providing zero cost) were then estimated; ii) the second part estimated the total accumulated costs, conditional on incurring any cost, by using the same set of three models applied in the one-part model group and described above. In this two-part set of analyses, the lognormal linear regression has not required to add 1 to the observed costs.

Cost ratios of patients with diabetes vs. those without diabetes were then estimated. Finally, estimated costs for patients with and without diabetes were calculated multiplying the expected probability of spending for health care by the estimated costs for people using health care (results shown only for gamma model).

Due to the uncertainty on the parametric assumption of the distributional forms, confidence intervals were calculated using a bootstrapping simulation process which is a data-based simulation method for assigning measures of accuracy to statistical estimates, used to produce inferences such as confidence intervals without knowing the type of distribution from which a sample has been taken. The bootstrap simulates what would happen if repeated samples of the population could be taken by constructing a number of resamples with replacement of the observed dataset. Standard errors of the parameter of interest are then estimated by the standard deviation of the parameter in the simulated samples. We extracted bootstrapping samples using a SAS System macro-generating 100 bootstrap random samples of patients [21].

To assess the performance of each model, the root mean square error (RMSE) was computed for each model. RMSE is a frequently used measure of the difference between values predicted by a model and values actually observed, providing the models are expressed in the same unit of measure, as in our case [20]. These individual differences are also called residuals, and the RMSE serves to aggregate them into a single measure of predictive power. Since the errors are squared before they are averaged, the RMSE gives a relatively high weight to large errors. A RMSE value closer to 0 is desirable.

All the analyses were conducted using the SAS System.

## Results

On July 31, 2003, 33,792 of the 896,915 Turin residents were classified as patients with diabetes. As previously described [14], the mean age of patients with diabetes was 67.7 years and 44.3 years for patients without diabetes. Twenty one percent of the cohort of patients with diabetes were treated with diet, 52.8 % with oral drugs and 26.2 % with insulin; 1,703 people (0.05 %) where considered as type 1 diabetes, while 32,089 people (99.5 %) as type 2 diabetes.

The overall cohort was characterized by the presence of a relevant subgroup with zero annual costs (21.5 % patients without diabetes and 1.3 % among patients with diabetes). Observed mean costs per person/year were € 3,348.6 (median € 1,314.9) in patients with diabetes and € 831.2 (median € 110.0) in patients without diabetes.

Table 1 shows the results of the applied multivariate statistical models, adjusted for age, gender and presence of diabetes. The first three rows refer to one part models. The OLS provided the effect of covariates in terms of absolute additive costs due to the presence of diabetes (€ 1,832). Lognormal and GLM provided a relative estimate of the effect: a 6-fold cost increase for diabetes versus non diabetes with the lognormal and a 2.6-fold increase with the GLM. The ratio between the estimated costs for patients with and without diabetes varies from 4.03 with the OLS to 4.69 with the GLM.

**Fig. 1:**
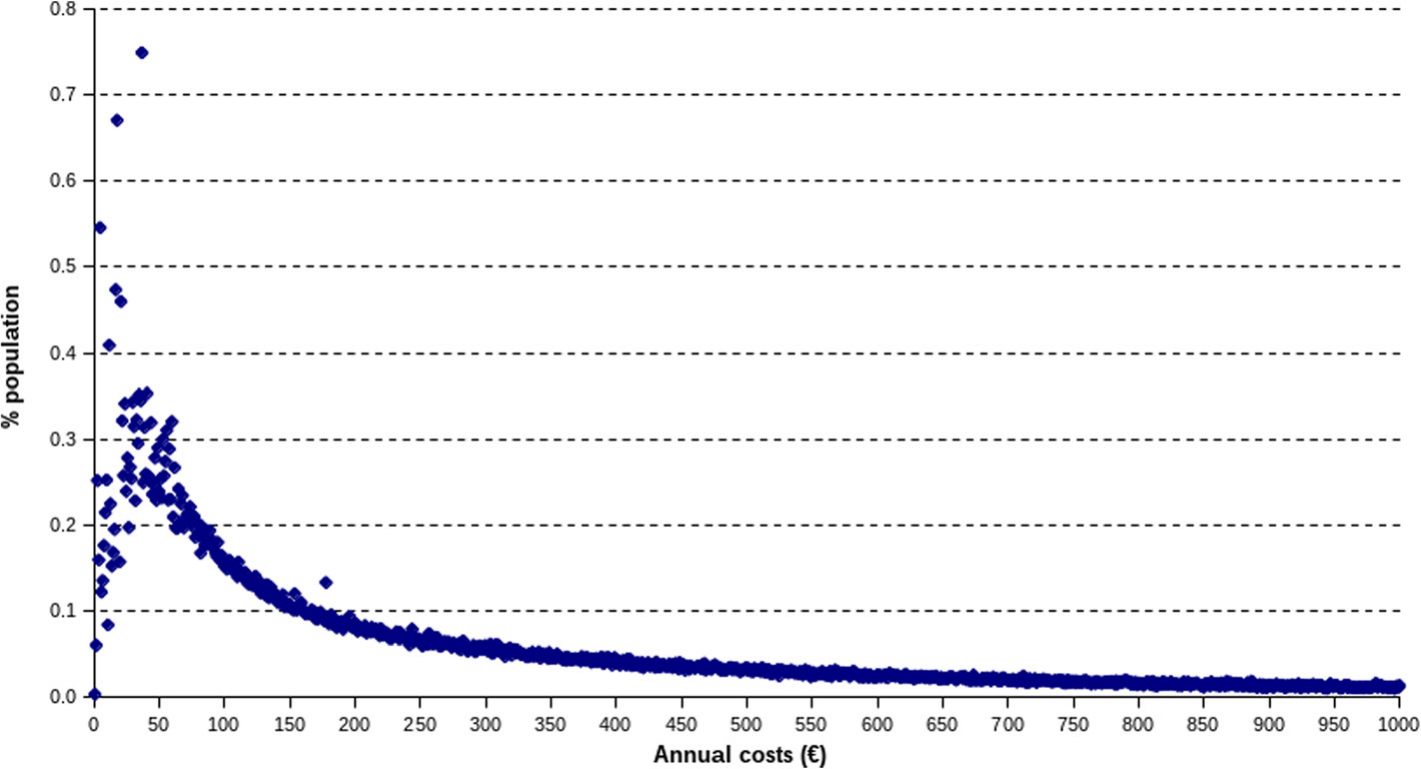
Distribution of annual healthcare costs among residents in the city of Turin (*n* = 896,915). Note. Excluded subjects with zero costs (20.8 %) and costs above 1000 euros

However, as shown in Fig. 1, the cost distribution was asymmetric (skewness coefficient: 14.3) and non-normal (Kolmogorov-Smirnov test: *p* < 0.01), due to the presence of zero cost observations and a relatively small proportion of people incurring extremely high costs. As a result, the assumption of normality in the distribution, required by the OLS model, did not hold and the coefficients estimated using this model could have been biased. The lognormal regression showed a greater probability of higher cost for patients with diabetes and higher estimated mean costs per person/year. The Gamma model provided final estimates close to the OLS albeit in presence of a statistically significant difference both in patients with and without diabetes, determining a higher cost ratio between the two groups (4.69).

The following four rows of Table 1 refer to the two part models. The first part of the model analyzed the probability of having had any costs (case with 0 costs) using a logistic regression. Results showed that the probability of incurring healthcare costs was higher for patients with diabetes than for those without diabetes (OR: 2,4; 95 % CI: 2.18–2.64). The pattern of estimated coefficients was similar to that obtained through the one part model, with the loglinear model showing higher estimated cost. The cost ratios estimated from the second part of the models referred to the treated patients only. The different percentage of zero cost observations in the two groups determines a relevant variation from the ratios estimated previously in the one part models.

The last row of Table 1 shows the estimated costs and cost ratio for the two-part gamma model, taking into account the probability of spending and the effective expenditure. The estimated cost ratio between patients with and without diabetes, considering the presence of zero cost observations in the two groups, was 4.10.

The RMSE was higher for loglinear model, whereas similar values were found for the other two models.

## Discussion

Our study provides a practical example of the relevance of using appropriate methods of analyzing costs of a chronic disease. Indeed, we showed that different methods provided substantially different estimated costs for patients with and without diabetes, and different costs ratios between them, ranging from 3.2 to 5.6. The range or variation of such estimated effects is relevant for health care planners; therefore careful choice of methods to analyze data is required before inferring results.

The increased availability of administrative date sources has largely increased the number of studies examining diseases-related costs, with the final aim of monitoring health care expenditure, identifying heterogeneities of expenditure among subgroups of patients and suggest strategies to improve resources. Moreover, data obtained from cost of illness studies are increasingly being incorporated into models used for assessing the cost-effectiveness of disease intervention, which assign incremental costs to specific subgroups of patients [22]. With respect to traditional epidemiologic research however, studies on costs of diseases are characterized by different methodological issues, which need to be appropriately handled to avoid biased results and wrong inferences about the distribution of patients’ health care costs. Healthcare cost distributions are typically affected by several criticisms, such as asymmetry, heteroscedasticity, presence of zero observations and censoring [13]. Although several methodological approaches have been identified in specialized literature in order to face properly such drawbacks, they are generally poorly known by clinicians. As an example, recent literature on diabetes costs is increasingly adopting non-traditional modeling methods [23–27]. However, the clinical audience is rarely skilled enough to understand the relevance of adopting appropriate methodological approaches.

Administrative data sources allows to manage large datasets and, as a general rule, when sample sizes are sufficiently large for the central limit theorem to exert itself, simple methods should be preferred. Nevertheless, also in large datasets, such as the one analyzed in the present study, the assumption of normality was not justified. Indeed, the cost distribution was strongly asymmetric and characterized by the presence of a relevant portion of subjects not using health services, particularly among patients without diabetes.

When applying different modeling approaches to disease costs, results show certain variability in the coefficient estimation because of the nature of the model, the units of measurement and the relative technique of retransformation. Moreover, the determination of the best performing model was not straightforward.

In our illustrative analysis, the loglinear model overestimated the effects with a low model precision. OLS regression holds on the assumption of normality, which was not supported by the skewness of the costs distribution of our data. Estimated costs were slightly underestimated among patients with diabetes compared with models that take into account the asymmetry of the distribution. Finally, the one part model ignored the information related to the zero costs observations.

Consistently, with application in other field of analysis [28–32], the evaluation of the best model for cost estimation of diabetes is not immediate.

Cost distribution characteristics and the objectives of the study should be fine-tuned to define the analysis plan.

If the study is focused on the analysis of health care system for policy planning, the two-part models should be preferred, because it makes it possible to quantify the global propensity to use healthcare resources, including subjects at zero costs.

If the focus is the estimation of the effect on cost of single covariates, − such as age, comorbidities, setting of care- a proper modelling of the observed positive costs is acceptable and easily interpretable.

## Conclusion

This study shows that costs estimates of a chronic disease vary considerably depending on the statistical method employed. Researchers involved in cost analyses as well as the potential users of the study results (clinicians and health care planners) should be aware of the impact of methodological choices on final results and interpretation.
